# Isolated Aneurysms of the Great Saphenous Vein: A Case Series and Review of the Literature

**DOI:** 10.7759/cureus.70505

**Published:** 2024-09-30

**Authors:** Alex Gidaya, Evan J Ryer, Joseph Allen, Isaiah Chandra, Anthony J Lewis, James R Elmore, Gregory G Salzler

**Affiliations:** 1 Department of Vascular and Endovascular Surgery, Geisinger Medical Center, Danville, USA

**Keywords:** great saphenous vein, saphenous aneurysm, vascular surgery, venous aneurysm, venous disease, venous varicosities

## Abstract

Great saphenous vein aneurysms (GSVA) represent a rare yet clinically significant condition, often misdiagnosed due to their infrequent presentation and resemblance to more common inguinal pathologies. This case series examines five instances of GSVA, emphasizing the diagnostic challenges, surgical interventions, and postoperative outcomes. Patients presented with varying symptoms, including groin masses and lower extremity pain, which were initially misattributed to other conditions. Imaging techniques, primarily venous duplex ultrasound, played a crucial role in identifying the aneurysms and guiding surgical planning. Surgical treatment, including aneurysm excision and ligation, was performed in all cases, with most patients experiencing uncomplicated postoperative courses. However, the risk of recurrence and thromboembolic events, such as pulmonary embolism, remains a concern, particularly in cases involving more proximal aneurysms near the deep venous system. Despite the success of surgical intervention, there is no consensus on the management of asymptomatic GSVA, nor are there standardized treatment guidelines. This case series highlights the need for heightened awareness among clinicians regarding GSVA, the importance of accurate diagnosis, and the consideration of prompt surgical treatment to prevent severe complications. Further research is needed to establish clear guidelines for managing both symptomatic and asymptomatic GSVA, particularly in relation to thromboembolic risk.

## Introduction

Venous aneurysms of the lower extremity are relatively rare vascular anomalies that can be classified into two primary categories: superficial and deep venous aneurysms. The superficial venous system includes the great saphenous vein (GSV) and the small saphenous vein (SSV), while the deep venous system comprises larger, more central veins, such as the femoral, popliteal, and tibial veins [1-6]. Of these, deep venous aneurysms, particularly popliteal venous aneurysms, are more frequently encountered in clinical practice [7-9]. These aneurysms are clinically significant due to their well-documented association with serious complications such as pulmonary embolism, which can be life-threatening if not promptly diagnosed and treated.

In contrast, superficial venous aneurysms are less common and often pose a greater diagnostic challenge. Despite their rarity, they carry significant clinical implications, as they can mimic other more prevalent inguinal pathologies, leading to delays in accurate diagnosis and appropriate management [3,10-14]. Specifically, large superficial venous aneurysms, particularly those occurring in the inguinal region, may present with symptoms such as localized pain, swelling, and lower extremity edema. These symptoms are frequently mistaken for other conditions, such as inguinal hernias or lymphadenopathy, which can further complicate the clinical picture and delay effective treatment.

The GSV is the most affected vein in cases of superficial venous aneurysms. Although they are often underrecognized, GSV aneurysms carry a considerable risk of significant complications, including recurrent pulmonary embolism [6]. Notably, such embolic events have been reported even in patients who are receiving anticoagulation therapy, underscoring the potential severity of this condition. The presence of a GSV aneurysm should prompt careful consideration of the risks and benefits of various treatment options, including surgical intervention, to prevent further thromboembolic complications.

Despite the potential for serious outcomes, there are currently no standardized guidelines for the management of venous aneurysms in the lower extremities, with the most recent 2023 Society for Vascular Surgery/American Venous Forum/American Vein and Lymphatic Society clinical practice guidelines only able to make an ungraded consensus statement [15]. The existing literature on the subject is limited, particularly regarding the clinical presentation and optimal treatment strategies for superficial venous aneurysms. This lack of robust data presents a challenge for clinicians tasked with managing these cases, as decisions often need to be made based on individual patient factors and the specific characteristics of the aneurysm.

Given the scarcity of comprehensive studies and the significant clinical implications associated with venous aneurysms of the lower extremities, there is a clear need for more research and consensus in this area. The purpose of this report is to contribute to the existing body of knowledge by presenting a case series of great saphenous venous aneurysms and reviewing the current literature. Through these efforts, we aim to provide insights into the clinical presentation, diagnostic challenges, and potential treatment options for this rare but important pathology. Ultimately, we hope to offer a concise framework that can guide clinicians in the effective management of great saphenous venous aneurysms, thereby improving patient outcomes and reducing the risk of severe complications.

## Case presentation

Case series

Case 1

**Figure 1:**
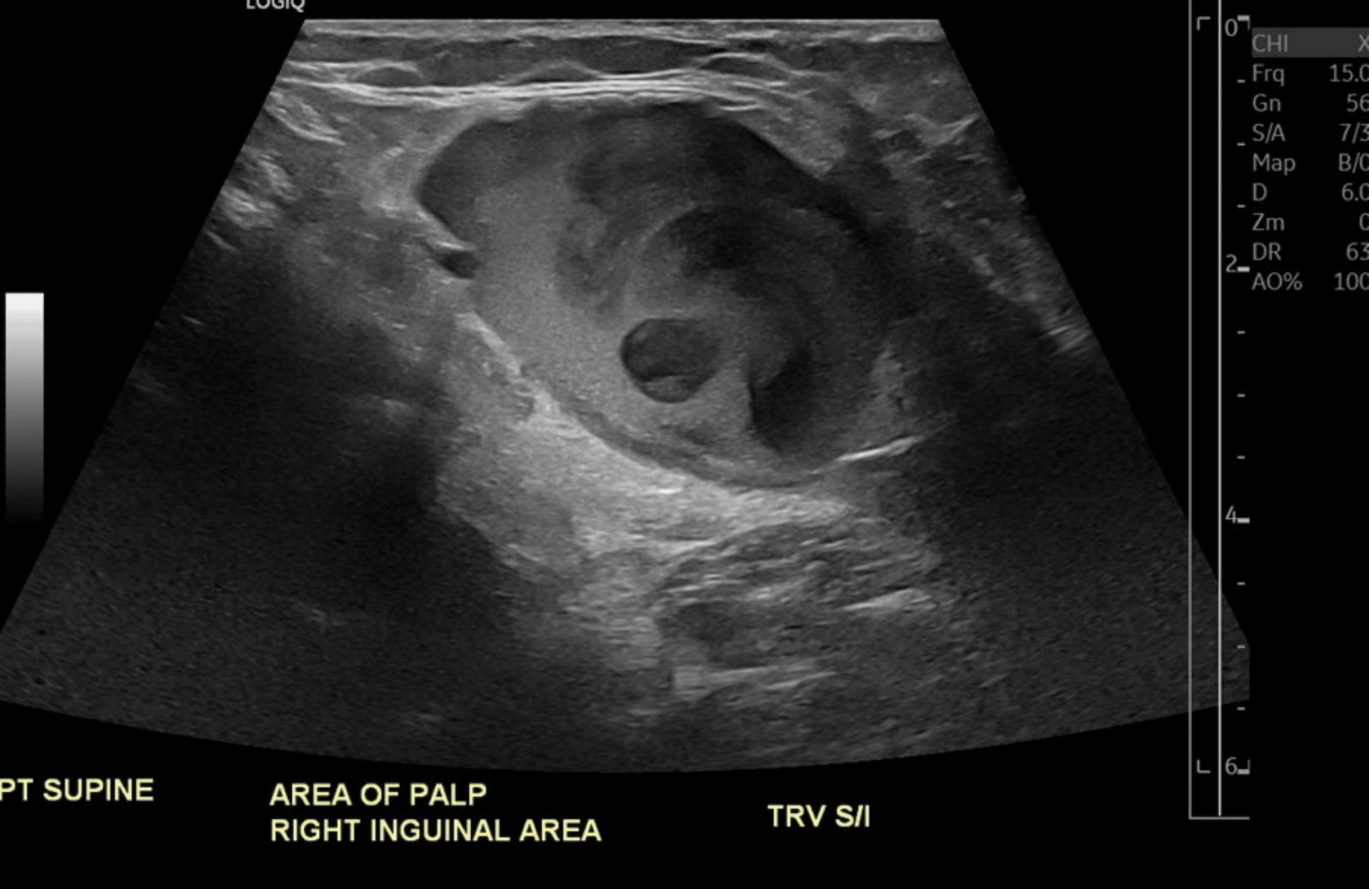
Abdominal ultrasound revealing a cystic structure filled with echogenic material, consistent with the thrombus

A 44-year-old obese male presented with a slow-growing, soft, non-tender, and reducible mass in the right groin, which had been present for several years. Previous imaging studies had identified the mass as an incidental finding, initially thought to be an enlarged lymph node. However, a recent evaluation by his primary care physician raised concerns about whether the mass was an enlarged lymph node or a right inguinal hernia. An abdominal ultrasound was performed, revealing a cystic structure filled with echogenic material consistent with a thrombus (Figure 1).

**Figure 2:**
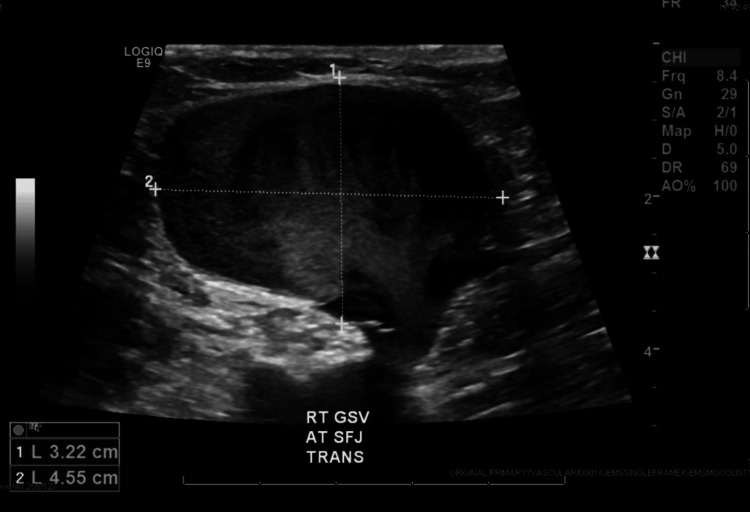
Venous duplex ultrasound identifying the mass as a 3.2 x 4.6 cm aneurysm of the proximal right great saphenous vein, adjacent to the saphenofemoral junction

Further investigation with a dedicated venous duplex ultrasound identified the mass as a 3.2 x 4.6 cm aneurysm of the proximal right great saphenous vein, located adjacent to the saphenofemoral junction (Figure 2).

**Figure 3:**
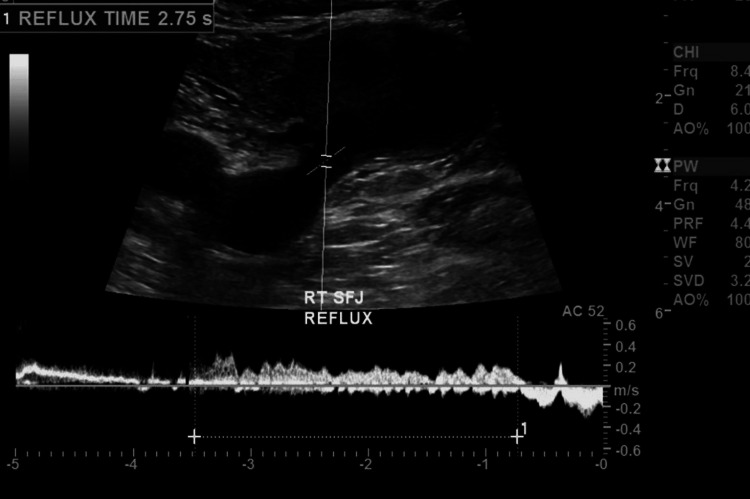
Venous duplex demonstrating venous reflux within the saphenofemoral junction along with great saphenous vein aneurysm

**Figure 4:**
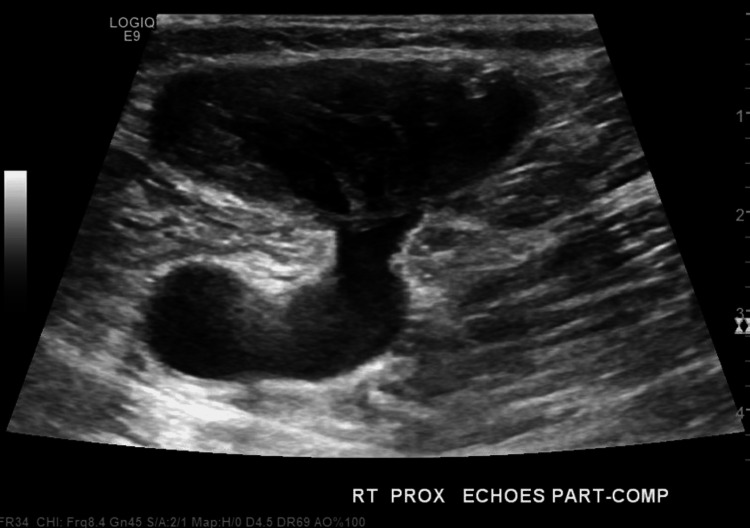
Venous duplex also demonstrating low flow within the aneurysm sac and thrombus encroaching on the saphenofemoral junction

The ultrasound also detected venous reflux within the common femoral vein, saphenofemoral junction, and great saphenous vein, as well as low flow within the aneurysm sac with thrombus encroaching on the saphenofemoral junction (Figures 3-4).

The patient had a history of chronic bilateral lower extremity varicose veins and had recently developed right lower extremity superficial thrombophlebitis, for which he was treated with a 45-day course of rivaroxaban (Xarelto). Prior to this episode, his varicosities had been asymptomatic.

Given the significant thrombus within the aneurysm despite anticoagulation therapy and the associated risk of thromboembolism, the decision was made to surgically excise the aneurysm. An oblique incision was made over the aneurysm, proximal to the great saphenous vein at the saphenofemoral junction. The aneurysm was carefully dissected free from surrounding tissues, and several branches were ligated. Clamps were placed at the neck of the aneurysm at the saphenofemoral junction and distally on the great saphenous vein. The aneurysm was then excised, and both the proximal saphenofemoral junction and the distal saphenous vein were oversewn with running 5-0 Prolene suture in two layers.

The patient's postoperative course was uncomplicated. Follow-up venous duplex ultrasound showed no evidence of deep vein thrombosis (DVT), and the patient was maintained on anticoagulation therapy for three months.

Case 2

**Figure 5:**
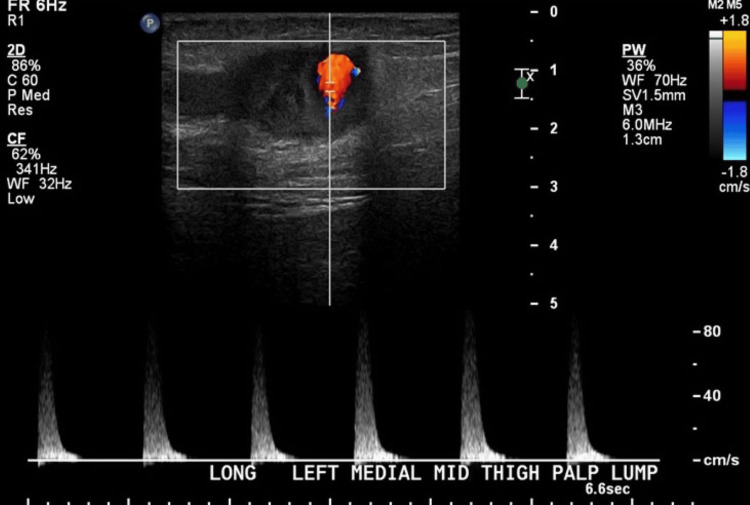
Left lower extremity ultrasound revealing a complex cystic structure with luminal blood flow and surrounding thrombus, raising concerns for an arteriovenous fistula

A 71-year-old male, with a past medical history of coronary artery disease and atrial fibrillation, managed with apixaban (Eliquis), presented with a mass in the left medial thigh. The patient reported first noticing the mass approximately two weeks prior but experienced no associated pain or discomfort. An initial ultrasound of the left lower extremity revealed a complex cystic structure with luminal blood flow and surrounding thrombus, raising concerns for an arteriovenous fistula (Figure 5).

**Figure 6:**
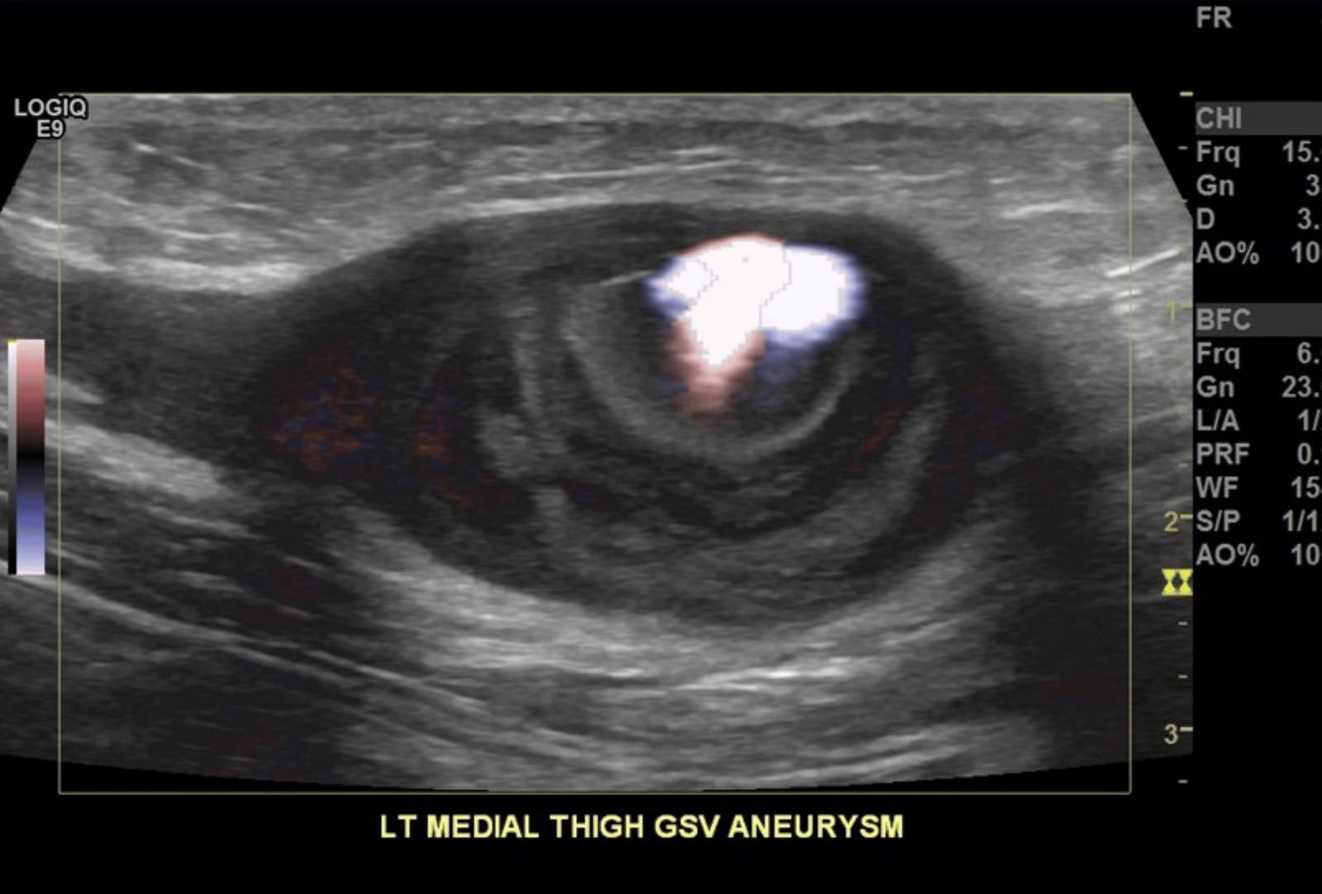
Venous duplex evaluation ultrasound identified a 3.6 x 2.1 x 2.5 cm aneurysm of the left great saphenous vein, with the thrombus extending proximally

Further evaluation with a venous duplex ultrasound identified a 3.6 x 2.1 x 2.5 cm aneurysm of the left great saphenous vein, with a thrombus extending proximally (Figure 6).

The patient subsequently underwent surgical excision of the left great saphenous vein aneurysm. A longitudinal incision was made over the mass along the length of the great saphenous vein at the proximal thigh. The aneurysm was carefully dissected, and the great saphenous vein was ligated proximally and distally. The aneurysm, containing a significant amount of thrombus, was completely excised.

The patient’s postoperative recovery was uncomplicated, and he resumed anticoagulation 24 hours post-surgery without any issues.

Case 3

**Figure 7:**
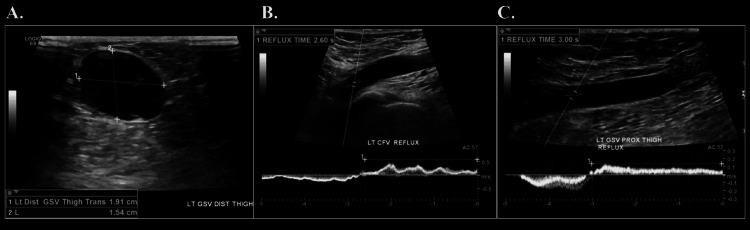
Venous duplex ultrasound revealing a 2 cm aneurysm of the left great saphenous vein (A), along with reflux at the common femoral vein (B) and proximal great saphenous vein (C)

A 39-year-old female, with a long-standing history of left lower extremity varicose veins, present since childhood, presented with worsening pain and swelling over the past five years. She also reported a lump at the distal medial thigh, which had been noticeable since the birth of her first child several years earlier. The lump became more prominent and painful with prolonged standing. A venous duplex ultrasound revealed a 2 cm aneurysm of the left great saphenous vein, along with reflux at the common femoral and great saphenous veins (Figure 7).

This patient underwent ligation of the venous aneurysm proximally and distally, followed by complete excision. The remaining left great saphenous vein underwent radiofrequency ablation accompanied by stab avulsion of multiple branch varicosities. A venous duplex performed at the end of the procedure confirmed that the treated segment of the great saphenous vein was non-compressible and without flow, while the normal flow was preserved in the left common femoral vein.

The patient’s postoperative course was uncomplicated. Follow-up imaging showed no evidence of DVT, and her symptoms resolved.

Case 4

**Figure 8:**
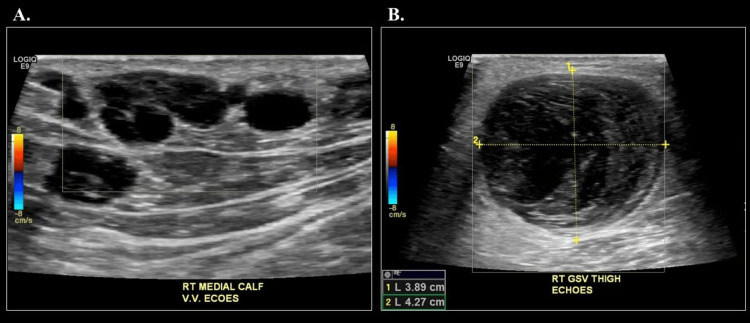
Venous duplex ultrasound revealing thrombophlebitis of superficial branch varicosities in the right medial calf (A) and a 4.3 cm aneurysm of the right great saphenous vein in the distal thigh (B)

A 66-year-old male with a history of unprovoked DVT in the left lower extremity, managed with warfarin, presented with pain and erythema in the right lower extremity. A venous duplex ultrasound revealed thrombophlebitis of superficial branch varicosities in the right medial calf and a 4.3 cm aneurysm of the right great saphenous vein in the distal thigh (Figure 8).

There was no flow within the great saphenous vein at the distal thigh and proximal calf, and the patient reported having a lump at the distal medial thigh for about four years, with no recent changes in size, pain, or discomfort.

Intraoperative venous duplex ultrasound confirmed a patent, large proximal right great saphenous vein measuring approximately 4 cm in diameter. The vein's size at the proximal thigh was too large for endovenous ablation, so the patient underwent high ligation and stripping of the right great saphenous vein, along with excision of the aneurysm. To accomplish this, an incision was made over the aneurysm at the distal medial thigh, and the aneurysm was dissected, clamped proximally and distally, and completely excised. The distal remnant vein was oversewn with a 5-0 Prolene suture, while the proximal remnant vein remained clamped. Next, an oblique incision was made at the right groin over the saphenofemoral junction, where the proximal saphenous vein, of normal caliber near the junction, was identified and dissected. The proximal branches were ligated with 2-0 silk ties. A clamp was placed on the proximal portion of the great saphenous vein, which was then sharply divided, and the stump oversewn with 5-0 Prolene. A vein-stripping catheter was passed through the great saphenous vein, and the vein was removed by pulling the catheter through the thigh. Pressure was applied to the thigh for hemostasis, and the wound was closed in multiple layers.

Postoperatively, the patient experienced pain and swelling in the right medial thigh, which gradually resolved. The remainder of his recovery was uncomplicated.

Case 5

**Figure 9:**
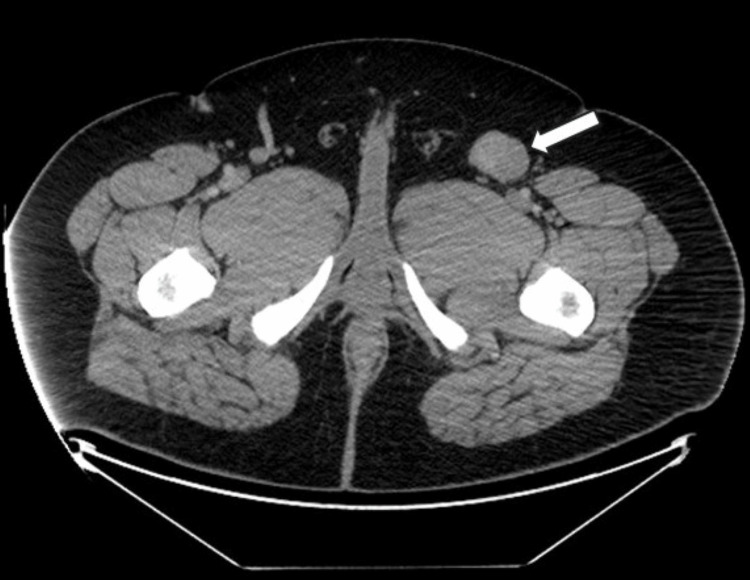
CT scan of the abdomen and pelvis revealing an indeterminate mass-like density in the left great saphenous vein, measuring 3.8 x 2.8 cm

A 22-year-old obese male presented to his primary care provider with a several-month history of left flank pain radiating to the groin. On examination, no palpable mass was found, but the patient exhibited tenderness in the left groin. A CT scan of the abdomen and pelvis revealed an indeterminate mass-like density in the left great saphenous vein, measuring 3.8 x 2.8 cm in diameter and 6.5 cm in length (Figure 9).

**Figure 10:**
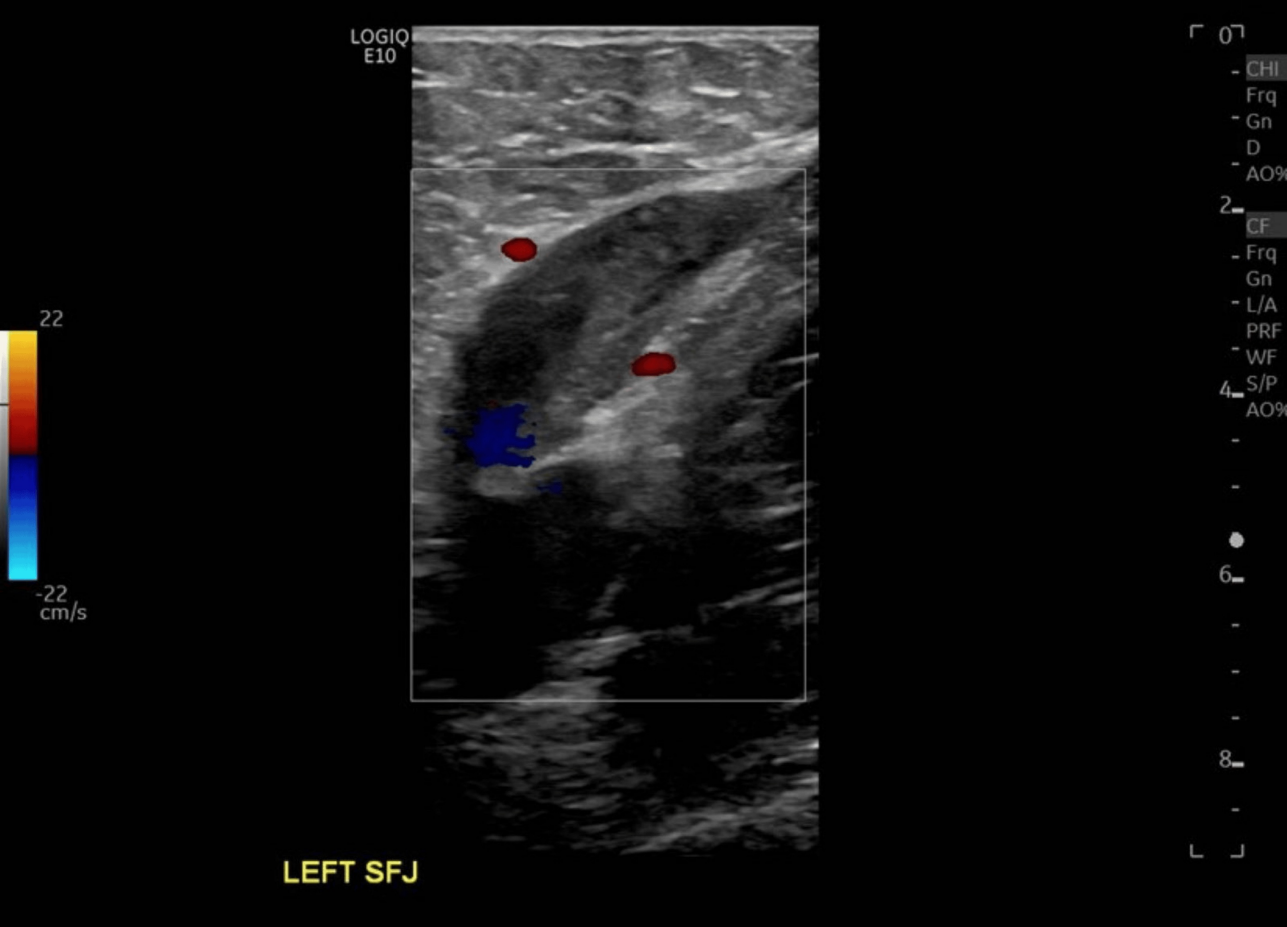
Venous duplex ultrasound demonstrating focal dilation of the proximal left great saphenous vein with thrombus formation, laminated flow, and decreased compressibility

This finding was confirmed by venous duplex ultrasound, which demonstrated focal dilation of the proximal left great saphenous vein with thrombus formation, laminated flow, and decreased compressibility (Figure 10).

Given the significant clot burden within the aneurysm and the risk of pulmonary embolism, the patient underwent surgical excision of the left great saphenous vein aneurysm. A longitudinal incision was made in the left proximal medial thigh. The aneurysm was found to be contiguous with a large common femoral vein. The aneurysm was clamped at the junction with the common femoral vein and 7 cm distally, where the vein was of normal caliber. The aneurysm was circumferentially dissected and excised. The distal aspect was oversewn with a 4-0 Prolene suture, and the common femoral vein was plicated at the proximal clamp site, with the defect also oversewn with a 4-0 Prolene suture.

The patient's immediate postoperative course was uncomplicated. His incisions healed well, and he was advised to continue systemic anticoagulation for six months postoperatively. However, the patient declined further follow-up beyond his one-month postoperative visit.

**Figure 11:**
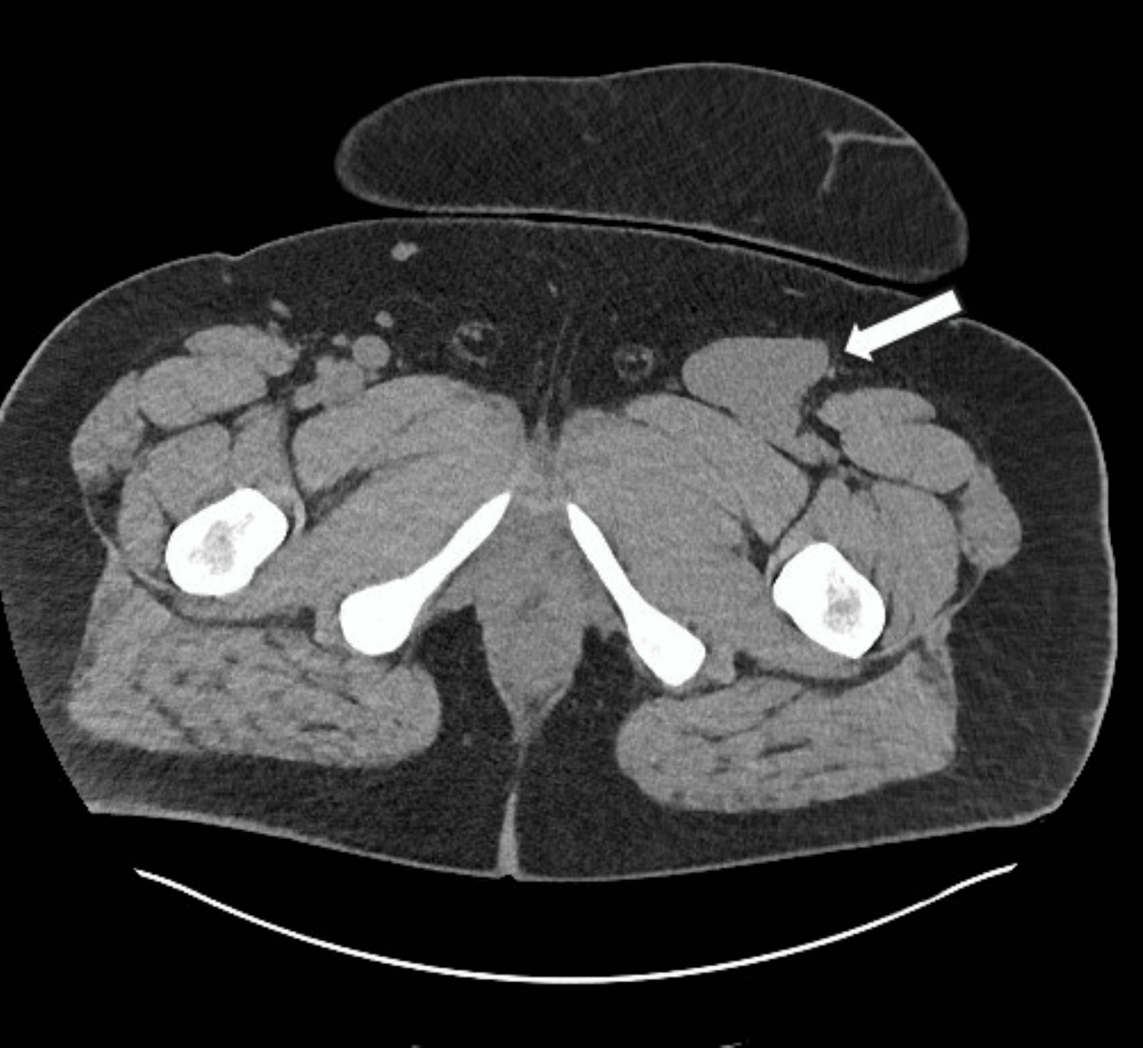
Non-contrast CT scan of the abdomen and pelvis showing a large isodense structure confluent with the left common femoral and great saphenous veins, raising suspicion for a recurrent aneurysm or pseudoaneurysm at the plication suture line

Two years later, the patient returned to the emergency department with a two-month history of left groin pain. He was referred to general surgery with concerns about a symptomatic left inguinal hernia. A non-contrast CT scan of the abdomen and pelvis showed a large isodense structure confluent with the left common femoral and great saphenous veins, raising suspicion for a recurrent aneurysm or pseudoaneurysm at the plication suture line (Figure 11).

**Figure 12:**
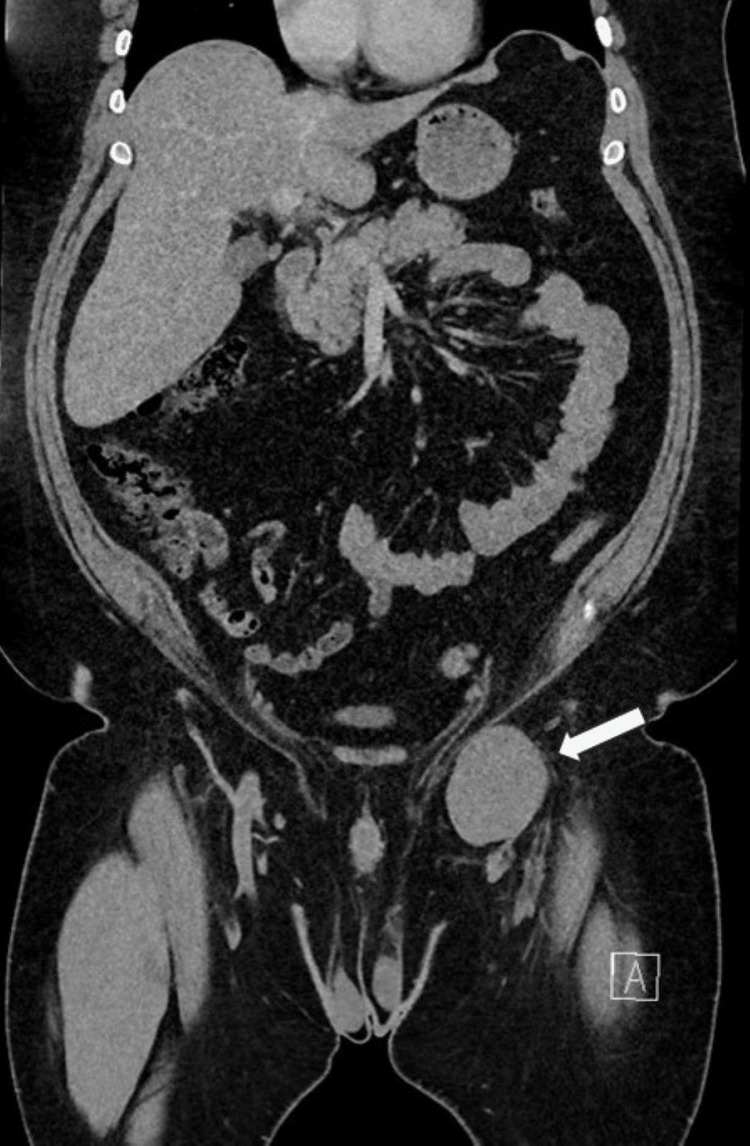
CT scan of the abdomen and pelvis with the venous phase imaging revealing a left common femoral vein aneurysm measuring 7.2 x 5.2 cm

The patient was referred to vascular surgery, where he presented with left groin pain but no palpable mass. Notably, he had gained 25 lbs. since his last visit, with a BMI of 52 kg/m². A CT scan of the abdomen and pelvis with venous phase imaging revealed a left common femoral vein aneurysm measuring 7.2 x 5.2 cm (Figure 12).

No thrombus was evident within the aneurysm, so anticoagulation was not initiated. The patient was scheduled for follow-up in two months to plan for resection and reconstruction of the left common femoral vein aneurysm. In the meantime, he was referred to Nutrition for weight loss assistance.

**Figure 13:**
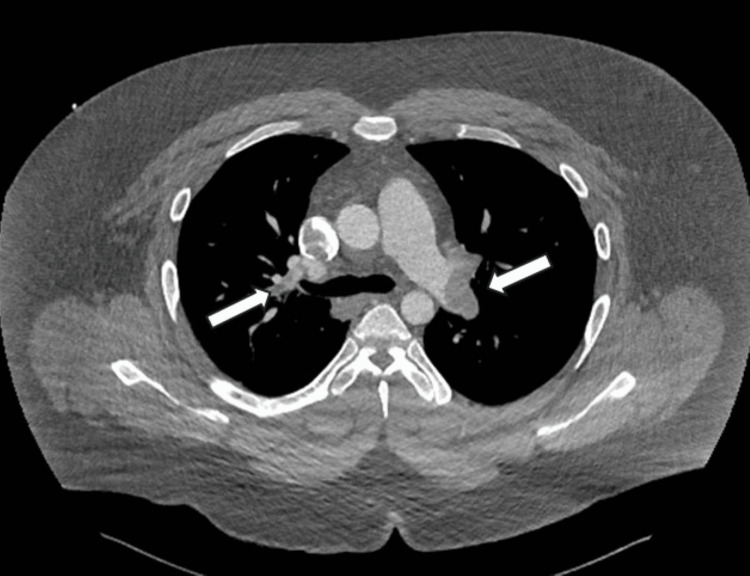
CTA chest revealed massive bilateral pulmonary emboli with evidence of right heart strain

Several weeks later, the patient presented to the emergency department with acute, severe shortness of breath. A CTA chest revealed massive bilateral pulmonary emboli with evidence of right heart strain (Figure 13).

Anticoagulation was started, and the patient was transferred to the intensive care unit. Despite aggressive measures, including VA-ECMO and CRRT, the patient’s condition rapidly deteriorated. He experienced a cardiac arrest but achieved a return of spontaneous circulation (ROSC) after 60 minutes. However, despite maximum-dose pressors and intensive treatment, the patient developed worsening multiorgan failure. Given the poor prognosis, the family opted for comfort measures, and the patient passed away shortly thereafter.

## Discussion

Great saphenous venous aneurysms are an uncommon clinical finding, with most insights derived from isolated case reports and a limited number of case series. The etiology of these aneurysms involves both congenital and acquired factors that affect the vessel wall. Congenital venous aneurysms are typically due to developmental deficiencies, characterized by the loss of smooth muscle and elastic fibers, resulting in varying degrees of wall thinning and compromised vessel integrity [5]. Acquired venous aneurysms are more commonly linked to conditions such as chronic venous insufficiency, trauma, or degeneration of allograft bypasses [7]. Chronic venous insufficiency, in particular, is frequently associated with these aneurysms.

Chronic venous insufficiency often results from reflux through incompetent venous valves, leading to increased venous pressure or hypertension. This condition subsequently causes smooth muscle hypertrophy, thickening of the intima, and breakdown of collagen. Both venous aneurysms and varicose veins show elevated expression of matrix metalloproteinases within endothelial and smooth muscle cells, which contribute to the degradation of elastic fibers and promote aneurysmal dilation of the vessel wall [1,5]. Furthermore, there is a notable correlation between large venous aneurysms and body mass index (BMI), with obese individuals exhibiting higher intra-abdominal pressure that exacerbates venous hypertension and consequently increases the risk for aneurysm formation [8,9].

Currently, there is no universally accepted size criterion for venous aneurysms. Traditionally, these aneurysms are described as localized areas of venous dilation that are distinct from varicose veins and not associated with pseudoaneurysms or arteriovenous fistulas [9]. Some sources suggest defining an aneurysm as a dilation at least 1.5 times the diameter of the contiguous vein, a threshold adapted from arterial aneurysm definitions [3,8]. However, this measure is not widely adopted or standardized within the venous system.

Despite the lack of a standardized size criterion, there is a well-established classification system for superficial venous aneurysms based on their anatomical location relative to deep venous structures. Pascarella et al. [8] proposed a classification system for superficial venous aneurysms. In this system, type I aneurysms are located at the proximal GSV, distal to the subterminal valve, without involving the saphenofemoral junction. Type II aneurysms are found in the mid or distal portions of the GSV. Type III includes both type I and type II aneurysms in the same extremity. Lastly, type IV aneurysms are located in the small saphenous vein.

Bush et al. [1] later refined this system. They defined type Ia aneurysms as those involving the saphenofemoral junction and type Ib aneurysms as located distal to the subterminal valve. Types IIIa and IIIb describe aneurysms with varying degrees of saphenofemoral junction involvement. Type IVa aneurysms involve the saphenopopliteal junction, while type IVb refers to more distal lesions. Types Va and Vb represent aneurysms in the proximal and distal anterior accessory saphenous vein, respectively, and type VI is for aneurysms that do not fit into any of the other categories.

Venous duplex ultrasound remains the primary non-invasive diagnostic tool for identifying lower extremity venous aneurysms. It provides detailed information on aneurysm size, color flow function, and its relationship to the subterminal valve or deep venous system [12]. Computed tomography (CT) is also valuable, particularly for surgical planning, and can help detect thrombus within the aneurysm. While some authors have suggested venography prior to surgical intervention [10,11], adjunctive imaging modalities such as CT, magnetic resonance imaging (MRI), and venography are typically reserved for cases where duplex ultrasound results are ambiguous [2].

For symptomatic superficial venous aneurysms, surgical intervention is often required, as anticoagulation alone does not address compressive symptoms or prevent recurrent thromboembolic complications [9]. The surgical approach to superficial venous aneurysms is generally less complex than that for deep venous aneurysms because restoring venous continuity is not necessary. Simple ligation and excision of the aneurysm are usually sufficient. However, for proximal aneurysms involving the saphenofemoral junction or common femoral vein, more elaborate procedures such as tangential aneurysmectomy with lateral venography or aneurysm resection with vein grafts may be necessary [9]. Endovenous ablation is generally not feasible due to the aneurysm's location and size [2].

Guidelines for managing asymptomatic superficial venous aneurysms are lacking, and the relationship between aneurysm size and thromboembolic risk remains unclear [9]. There have been reports of pulmonary embolism resulting from great saphenous venous aneurysms even in patients who were asymptomatic prior to diagnosis [6]. Therefore, selected asymptomatic patients, particularly those with high-risk proximal aneurysms or significant thrombus, may benefit from surgical intervention. The role of anticoagulation in this context is not well defined, though some reports suggest a postoperative anticoagulation regimen lasting three to six months [9]. Venous duplex ultrasound remains the preferred method for postoperative imaging, and the timing and frequency of follow-up should be tailored to the individual patient's needs and the surgeon’s discretion.

Lastly, it is paramount to discuss the manuscript on this topic from the Vascular Low-Frequency Disease Consortium (VLFDC). The "Contemporary Management and Outcomes of Peripheral Venous Aneurysms: A Multi-Institutional Study," published in the Journal of Vascular Surgery: Venous and Lymphatic Disorders in November 2022, provides a thorough analysis of current practices and outcomes related to peripheral venous aneurysms (PVAs) [14]. Through a multi-center approach, the study evaluates various treatment strategies, including surgical excision, ligation, and endovenous methods, and their effectiveness in managing symptomatic PVAs. The findings reveal that, while surgical interventions generally lead to favorable outcomes with significant symptom relief and low complication rates, there is notable variability in treatment protocols across different institutions. This variability highlights the need for standardized guidelines to ensure optimal patient care and outcomes. The study also emphasizes the importance of long-term follow-up to detect recurrences and evaluate the sustainability of treatment effects. These insights underscore the necessity for ongoing research to develop evidence-based recommendations and improve the overall management of PVAs.

## Conclusions

GSVA are rare but significant, often challenging to diagnose due to their resemblance to other inguinal and lower extremity conditions. This case series highlights the diagnostic complexity and emphasizes the crucial role of venous duplex ultrasound in confirming GSVA and guiding surgical decisions. Surgical excision and ligation were successful in all symptomatic cases, leading to uncomplicated recoveries. However, the risk of recurrence and thromboembolic events, especially with proximal aneurysms, underscores the need for careful management and ongoing vigilance.

The absence of standardized guidelines for asymptomatic GSVA management presents a challenge, necessitating individualized treatment based on factors such as thrombus presence and aneurysm location. Anticoagulation therapy remains important postsurgery, yet its role in asymptomatic cases is unclear. Increased awareness, improved diagnostic practices, and further research are essential to develop clear guidelines for managing both symptomatic and asymptomatic GSVA. Future studies should focus on refining treatment protocols and understanding thromboembolic risks to enhance patient outcomes.
